# A Case of Neuromyelitis Optica: Puerto Rican Woman with an Increased Time Lag to Diagnosis and a High Response to Eculizumab Therapy

**DOI:** 10.1155/2022/4311382

**Published:** 2022-02-18

**Authors:** Ramón Vega, Benjamín González, Kiara Ortiz, Viviana Martínez, David Carmona, Ivonne Vicente, Javier Chapa, Ángel Chinea

**Affiliations:** ^1^Puerto Rico Multiple Sclerosis Foundation, Centro Internacional de Mercadeo, 100 Carr. 165, Torre 1, Suite 403, Guaynabo, PR 00968, USA; ^2^San Juan MS Center, Centro Internacional de Mercadeo, 100 Carr. 165, Torre 1, Suite 306, Guaynabo, PR 00968, USA; ^3^San Juan Bautista School of Medicine, Urb. Turabo Garden Carr. 172, Caguas, PR 00725, USA; ^4^Neurologist, Neurophysiologist, Epileptologist, and Neuroimager, Centro Internacional de Mercadeo, 100 Carr. 165, Torre 1, Suite 311, Guaynabo, PR 00968, USA

## Abstract

A link between intractable hiccups, as the initial symptom, and a possible neuromyelitis optica spectrum disorder (NMOSD) diagnosis is confusing but vital and may not be made by health care providers (HCPs) if they are not aware of the 2015 NMOSD criteria. Early diagnosis and adequate treatment are essential to prevent disease progression. We report the case of a 46-year-old Puerto Rican female who presented intractable hiccups when she was 31 (in 2004). Almost 15 years passed since the initial symptom, and after two severe relapses, she received a formal NMOSD diagnosis in March 2019. Treatment started with rituximab 1000 mg IV in April 2019. However, a lack of response to treatment led to a switch to eculizumab therapy in August 2019. The patient had cervical and brain magnetic resonance imaging (MRI) conducted in June 2020, which depicted a remarkable decrease in swelling and hyperintensity within the cervical spinal cord with no enhancing lesions when compared with the first MRI from February 2019. In addition, the patient suffered no new relapses, an improvement regarding disability, and a reduction of the cervical spinal cord lesion size. Nonetheless, this substantial decrease does not occur on all NMOSD patients, but more awareness of the disease is needed, especially in Puerto Rico. This case illustrates the efficacy of eculizumab therapy and the importance of differentiating the clinical, histopathological, and neuroimaging characteristics that separate demyelinating autoimmune inflammatory disorders, such as NMOSD and multiple sclerosis (MS).

## 1. Introduction

Neuromyelitis optica (NMO) is a demyelinating autoimmune inflammatory disorder of the central nervous system (CNS) that primarily affects women [1]. It accounts for 0.37/100,000 to 4.2/100,000 cases in Latin America (including islands from the Caribbean) [2]. Additionally, neuromyelitis optica spectrum disorders (NMOSDs) are a clinically and radiologically defined group of CNS inflammatory autoimmune demyelinating diseases associated with a pathogenic antibody specific for the aquaporin-4 (AQP4) water channel [3, 4]. Clinical manifestation frequently begins with decreased visual acuity followed by paresthesia (numbness), dysesthesia (burning and electric shock sensations), and tonic spasms [5]. Characteristically, NMO patients present optic neuritis and transverse myelitis [1]. These symptoms resemble those commonly seen in multiple sclerosis (MS) [5]. However, these symptoms are present in a less severe manner in MS compared to NMO [6]. Neuroimaging, immunological, and histopathological characteristics that have been identified differentiate NMO from MS [7]. For this report, the terms NMO and NMOSD will imply the same condition and will thus be interchangeable.

## 2. Case Presentation

At the age of 31 (in 2004), this 46-year-old female presented intractable hiccups. As the initial symptom disappeared, not a single health professional delved deeper into its cause. She then presented with episodic pruritus (skin itching), dysesthesia (burning sensation), and paresthesia (numbness) on different parts of her body.

The patient describes having an episode of ophthalmalgia (eye pain), blurred vision, and epiphora (tearing) on the left eye in 2016. After an ophthalmologist's evaluation, she was referred for a neurologist's examination. Shortly after, she attended the emergency room and was admitted to a New York hospital. A neurologist ordered brain and cervical spinal cord magnetic resonance imaging (MRI) and spinal tap for cerebrospinal fluid (CSF) analysis to assess the situation. As a result, brain and cervical MRIs did not show lesions, and CSF was negative for oligoclonal bands. However, while hospitalized, the patient gradually lost a large percentage of her vision in the left eye. She was treated with corticosteroids and partially recovered. Based on the MRIs and CSF findings, the patient received an initial diagnosis of atypical optic neuritis. She was advised to stay vigilant if any symptoms recurred because these are typically seen in patients with multiple sclerosis. Following this initial diagnosis, she continued suffering from episodic paresthesias through the years.

**Figure 1:**
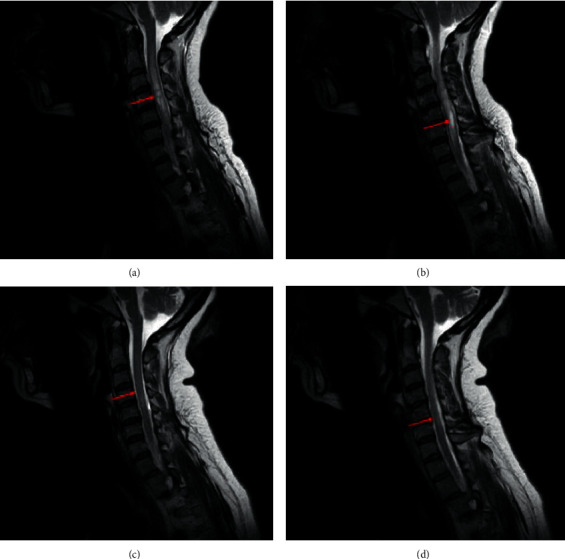
MRI of the cervical spinal cord-sagittal T2-weighed sequences ((a)–(d)). ((a), (b)) Sequences from February 2019 show an expansion of the cervical spinal cord involving a long segment from C1 through C7. ((c), (d)) Sequences from June 2020 show interval minimization in hyperintensity and swelling within the cervical spinal cord, and when compared to (a) and (b), no enhancing lesions are noted.

**Figure 2:**
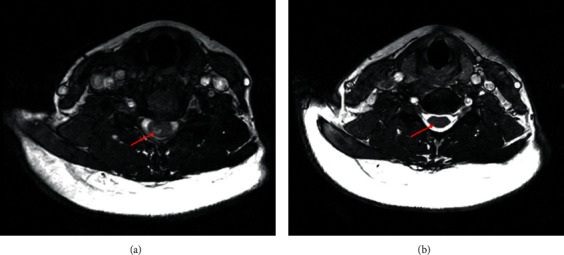
MRI of the cervical spinal cord-axial T2-weighed sequences ((a), (b)). (a) Sequence from February 2019 shows an abnormal signal intensity that involves the center of the cord and minimally extends to the cord's periphery. (b) Sequence from June 2020 shows an interval decrease in both swelling and hyperintensity.

Approximately 15 years after the hiccups presentation, in 2019, the patient had an episode of right hemibody paresthesia, Lhermitte's sign, and episodic abdominal distention. Following these symptoms, the patient moved to Puerto Rico and visited a neurologist. In February 2019, the neurologist ordered a new brain and cervical MRI and aquaporin-4 antibody (AQP4-IgG) cell-based assay (CBA) test. Further, she was treated with a 500 mg methylprednisolone IV for three days to decrease symptoms. A neuroimaging specialist interpreted the cervical MRI, and he reported that on the sequences without contrast, there was an expansion of the cervical spinal cord involving long segments from C1 through C7 (Figures 1 and 2). Upon administering IV contrast, the specialist concluded a rim enhancement with central nonenhancing cord lesion at the level of C5-C6 measuring approximately 1.5 cm (images not shown).

On the other hand, the brain MRI was described as unremarkable and showed no lesions. As a result, suggested diagnoses were transverse myelitis, neuromyelitis optica, multiple sclerosis, and low-grade astrocytoma. With a positive result for AQP4-IgG in the CBA, the patient formally received an NMOSD diagnosis. The neurologist recommended initial treatment with rituximab 1,000 mg IV monthly.

Rituximab therapy was administered in April and May 2019, but the patient was not responding to treatment. She presented Lhermitte's sign, gait instability, dizziness, and vertigo. The decision to discontinue rituximab was physician/patient-based. The patient did not want to continue since symptoms did not improve, and the treating neurologist concluded that the patient was not responding to treatment because she presented neurological signs that reflected a relapse. For this reason, rituximab treatment was discontinued in July 2019.

The treating neurologist suggested switching to 300 mg/ml of eculizumab every two weeks, and treatment began in August 2019. Overall, the patient responded well to eculizumab treatment. However, in February 2020 (6 months later), she presented persistent tremors on the right hemibody, right eye pain, and paresthesia in the feet and right hand. Consequently, intravenous methylprednisolone was used to treat these symptoms.

In March 2020, the patient temporarily stopped treatment due to fear of infection with COVID-19 and resumed once molecular and serological test results were negative. New brain and cervical MRIs were performed in June 2020. Compared to previous MRIs from February 2019 in a sagittal view (Figures 1(a) and 1(b)), the neuroimaging specialist concluded an interval decrease in swelling and hyperintensity within the cervical spinal cord (Figures 1(c) and 1(d)). Additionally, the axial view of the cervical spinal cord lesion from the February 2019 MRI (Figure 2(a)) is compared with the axial view of the June 2020 MRI (Figure 2(b)). Further, no enhancing lesions were noted in the most recent cervical MRI (when compared to the previous MRI). When brain MRIs were compared, no interval changes were observed.

## 3. Discussion

In 2006, NMO IgG was discovered, and the criteria for NMO diagnosis was revised [8]. Jarius et al. [8] established that, for an NMO diagnosis, the following criteria had to be met: optic neuritis, acute myelitis, and at least two of three supportive criteria. These criteria consisted of longitudinally extensive transverse myelitis (LETM, >3 vertebral segments), a brain MRI negative for MS criteria, and AQP4-IgG seropositive status [9].

More recently, in 2015, new criteria were established thanks to rapid advancements in NMO research. On these criteria, in addition to optic neuritis and acute myelitis, four other core clinical characteristics of NMO have been included: area postrema syndrome, acute brainstem syndrome, narcolepsy, or acute diencephalic clinical syndrome, and cerebral syndrome [9, 10]. In the case of patients seropositive for AQP4-IgG, only one core clinical characteristic must be met. On the other hand, for AQP4-IgG seronegative patients (a third of these patients are MOG-IgG positive), two core clinical characteristics and additional MRI features must be present [9].

Neuroanatomic locations of core clinical presentations include optic nerve, spinal cord, area postrema (dorsal medulla), diencephalon, brainstem, and cerebrum [9]. Although nonspecific cerebral lesions could be seen in the MRI of NMO patients at the onset, it has been demonstrated that NMO commonly spares the brain in the early stages. However, new nonspecific brain lesions (for NMO) develop in 60% of patients after they are diagnosed [11].

At the time of the hiccup presentation (2004), Wingerchuk NMO criteria were yet to be established at the time of the hiccup presentation [11]. Furthermore, hiccups may have been a confusing clinical feature for health care providers, due to their relation to multiple illnesses such as gastroesophageal reflux disease (GERD), myocardial infarction, brain ischemia, or stroke [12–14]. Even though the optic neuritis this patient suffered (2016) met with one core clinical characteristic of the 2015 NMO diagnostic criteria, the only supportive criterion was a brain MRI negative for MS. As for the cervical MRI, no lesions were seen in the 2016 MRI. With this said, if the neurologist had ordered AQP4-IgG testing, possible seropositivity would have confirmed the NMO diagnosis following the 2015 criteria.

In 2019, the patient suffered persistent right hemibody paresthesia (second severe relapse). In the search for an accurate diagnosis, the neurologist ordered cervical and brain MRIs. A new lesion seen in the cervical MRI yielded an essential supportive criterion that was not met with the initial cervical MRI performed in 2016. The neurologist then ordered an AQP4-IgG CBA to confirm an NMO diagnosis. A formal diagnosis arrived thanks to seropositivity for AQP4-IgG. This diagnosis came after having suffered two severe relapses (3 years apart) and a total of 3 key events when considering the intractable hiccups. It is essential to highlight that because AQP4-IgG testing was not parallel to the initial onset symptom, an association between the optic neuritis (suffered in 2016) and the event of intractable hiccups (12 years prior) cannot be drawn.

Treatment for neuromyelitis optica usually focuses on decreasing the inflammatory process characteristic of this condition [5]. For acute symptoms or flares, corticosteroids are the primary therapy, and plasmapheresis can be considered an alternative therapy for nonresponders to high doses of corticosteroids [8, 15]. Studies have shown that a combination of plasmapheresis and immunosuppressors decrease anti-AQP4 antibody serum levels [16, 17]. For long-term treatment, patients receive intravenous rituximab, which targets B cells [18, 19]. Other treatment options include methotrexate, mycophenolate mofetil, or mitoxantrone, but they are not considered initial treatment because of the significant side effects [20].

A recent study demonstrated the effectiveness of eculizumab (a humanized monoclonal antibody), which significantly reduced the risk of relapse compared to those (patients or subjects) that received a placebo [21]. In terms of its mechanism of action, eculizumab inhibits the terminal complement protein C5 and avoids its cleavage into C5a, proinflammatory, and C5b, which coordinates the formation of the membrane attack complex [22]. AQP4-IgG activates the complement cascade [23, 24]. This activation results in inflammation and the creation of the membrane attack complex. This complex is implicated in astrocyte destruction and neuronal injury but cannot be seen in experimental models in a complement inhibitor's presence [25]. In this case presentation, we observed a marked decrease in hyperintensity and swelling on the LETM lesion. Images show few residual scarring after presenting a lesion of that size (from C1 down to C7).

## 4. Conclusion

Intractable hiccups can be considered a possible initial manifestation of NMOSD but are usually not correctly identified due to its multiple causalities. This miss identification is what occurred with the patient we reported. Therefore, a considerable time lag to diagnosis could have been avoided with an essential association between intractable hiccups and NMOSD. This connection would have encouraged using the correct set of diagnostic tools (in this case, AQP4-IgG testing) needed to make a conclusive NMOSD diagnosis. For this reason, it is vital to create awareness, among healthcare providers, of the symptoms at onset (hiccups) and the diagnostic criteria of NMOSD. Additionally, as observed on the patient reported, initial treatment may not be effective immediately. Hence, it is essential to try other therapies that could help stop this aggressive disease's progression. We observed that after the switch to eculizumab therapy and approximately 11 months of treatment, MRI comparison revealed that the large lesion diminished significantly. The disease's progression decreased (even though there was a considerable time lag between initial presentation and diagnosis). This substantial decrease does not occur on all NMOSD patients, but more awareness of the disease is needed, especially in Puerto Rico.

## Data Availability

The patient consent form is available upon request. Postcontrast cervical spinal cord MRI sequences from February 2019 (images not shown) are available upon request.

## Abbreviations

MRI: Magnetic resonance imaging
NMO: Neuromyelitis optica
NMOSD: Neuromyelitis optica spectrum disorder
AQP4-IgG: Aquaporin-4 antibody
CBA: Cell-based assay
GERD: Gastroesophageal reflux disease
LETM: Longitudinally extensive transverse myelitis
MS: Multiple sclerosis
CSF: Cerebrospinal fluid.
